# Veterans have greater variability in their perception of binocular alignment

**DOI:** 10.1371/journal.pone.0209622

**Published:** 2018-12-26

**Authors:** Michael C. Schubert, Yoav Gimmon, Jennifer Millar, Kelly J. Brewer, Dale Roberts, Mark Shelhamer, Charles Rohde, Jorge M. Serrador

**Affiliations:** 1 Laboratory of Vestibular NeuroAdaptation, Department of Otolaryngology—Head and Neck Surgery Baltimore, MD, United States of America; 2 Department of Physical Medicine and Rehabilitation, Johns Hopkins University School of Medicine, Baltimore, MD, United States of America; 3 War Related Illness and Injury Study Center, Department of Veteran Affairs, NJ, United States of America; 4 Department of Neurology, Johns Hopkins University School of Medicine, Baltimore, MD, United States of America; 5 Bloomberg School of Public Health, Johns Hopkins University School of Medicine, Baltimore, MD, United States of America; 6 Department of Pharmacology, Physiology & Neuroscience, New Jersey Medical School, Rutgers School of Graduate Studies, Newark, NJ, United States of America; Taipei Veterans General Hospital, TAIWAN

## Abstract

**Introduction:**

A significant population of our wounded veterans suffer long-term functional consequences of visual deficit, disorientation, dizziness, and an impaired ability to read. These symptoms may be related to damage within the otolith pathways that contribute to ocular alignment. The purpose of this study was to compare perception of vertical and torsional ocular alignment between veterans and healthy controls in an upright and supine test position.

**Materials and methods:**

Veterans (n = 26) with reports of dizziness were recruited from the East Orange Veterans Administration Hospital. Healthy controls (n = 26) were recruited from both Johns Hopkins University and the East Orange VA. Each subject performed 20 trials each of a novel vertical and torsional binocular alignment perception test. Veterans underwent semicircular canal and otolith pathway function testing.

**Results:**

88% of the Veterans had an absent otolith response. Only the veterans had an abnormally large variability in perception of both vertical and torsional ocular alignment, and in both upright and supine position. Neither post-traumatic stress disorder, nor depression contributed to the misperception in binocular alignment.

**Conclusions:**

Our novel method of measuring vertical and torsional misalignment distinguishes veterans with dizziness from healthy controls. The high prevalence of absent otolith function seems to explain this result. Further studies are needed to better understand the fundamental mechanism responsible for the increased variability of perception of binocular alignment.

## Introduction

Brain injury via blast or blunt mechanisms disrupts multiple sensorimotor systems simultaneously and veterans from the Operation Iraqi Freedom/Operation Enduring Freedom campaigns report related physical, sensory, cognitive, and behavioral/emotional changes [1–44]. Typically, symptoms related to these damaged systems recover within weeks and significant improvement is often seen after three months [5]. However, a significant population of our wounded veterans suffer long-term functional consequences of visual deficit, postural and locomotor instability, disorientation, dizziness, and an impaired ability to read. Many of these symptoms are overlooked in patients with polytrauma [6]. Earlier descriptions of such symptoms reported a third of service members exposed to blast trauma had combined visual and hearing impairment, termed dual-sensory impairment [7,8]. However, more recent evidence suggests that within the population of these veterans exposed to traumatic brain injury (TBI), a clinical pattern of damage to the auditory, visual, and vestibular sensorimotor systems has emerged, which has collectively been given the name multi-sensory impairment (MSI) [9,10].

Evidence suggests nearly 20% of veterans diagnosed with the mild form of traumatic brain injury (mTBI) have MSI, as examined from a database of >13700 veterans [10]. Amongst a variety of predictors (i.e., older age, female gender, posttraumatic stress disorder), having a prior history of mTBI was the most robust for developing MSI. Among active duty service members, 15–25% deployed to Iraq or Afghanistan have sustained an mTBI during their tour, though other studies have reported a larger percentage (44%) [11–14]. Additionally, large numbers of US Gulf War era and OIF/OEF veterans with mTBI (>350K) are suffering MSI [15].

Intact vestibular function is essential for gaze and gait stability during rapid head movements, which is achieved via the vestibular systems’ exquisitely sensitive detection of angular and linear head motion. The vestibulo-ocular reflex (VOR) generates compensatory rotational and linear eye movements that are opposite in direction, but equal in magnitude, to the head motion. Additionally, spinal reflexes initiated from the vestibular afference ensure upright posture and righting responses. Most studies of vestibular damage in service members and veterans have measured the output of the horizontal semicircular canal, via the angular VOR. However, evidence suggests the utricular and saccular otolith end organs, not the semicircular canals (84% vs 29% respectively), are more vulnerable to blast-related injuries experienced in veterans [16].

The saccular maculae in particular may be more susceptible to injury as it is positioned close to the footplate of the stapes and thus subject to mechanical disruption [17, 18]. Damage to the utriculo-ocular pathway often causes the pathologic ocular tilt reaction (OTR). The OTR is a triad of signs that favor one side (i.e., damaged right utricle can cause rightward head tilt, the right eye to be lower than left eye, and the superior poles of both eyes to be rotated in roll toward the right). Central lesions in the lower brainstem (medulla and the peripheral vestibular afferent pathways) can cause an ipsilateral OTR while lesions in the upper brainstem may cause a contraversive OTR [19–21].Therefore, patients with otolith pathology may have abnormal vertical or torsional ocular alignment; known to differ when positioned supine vs upright. A skew identified in sitting due to an otolith asymmetry may decrease when positioned supine [22].

**Fig 1:**
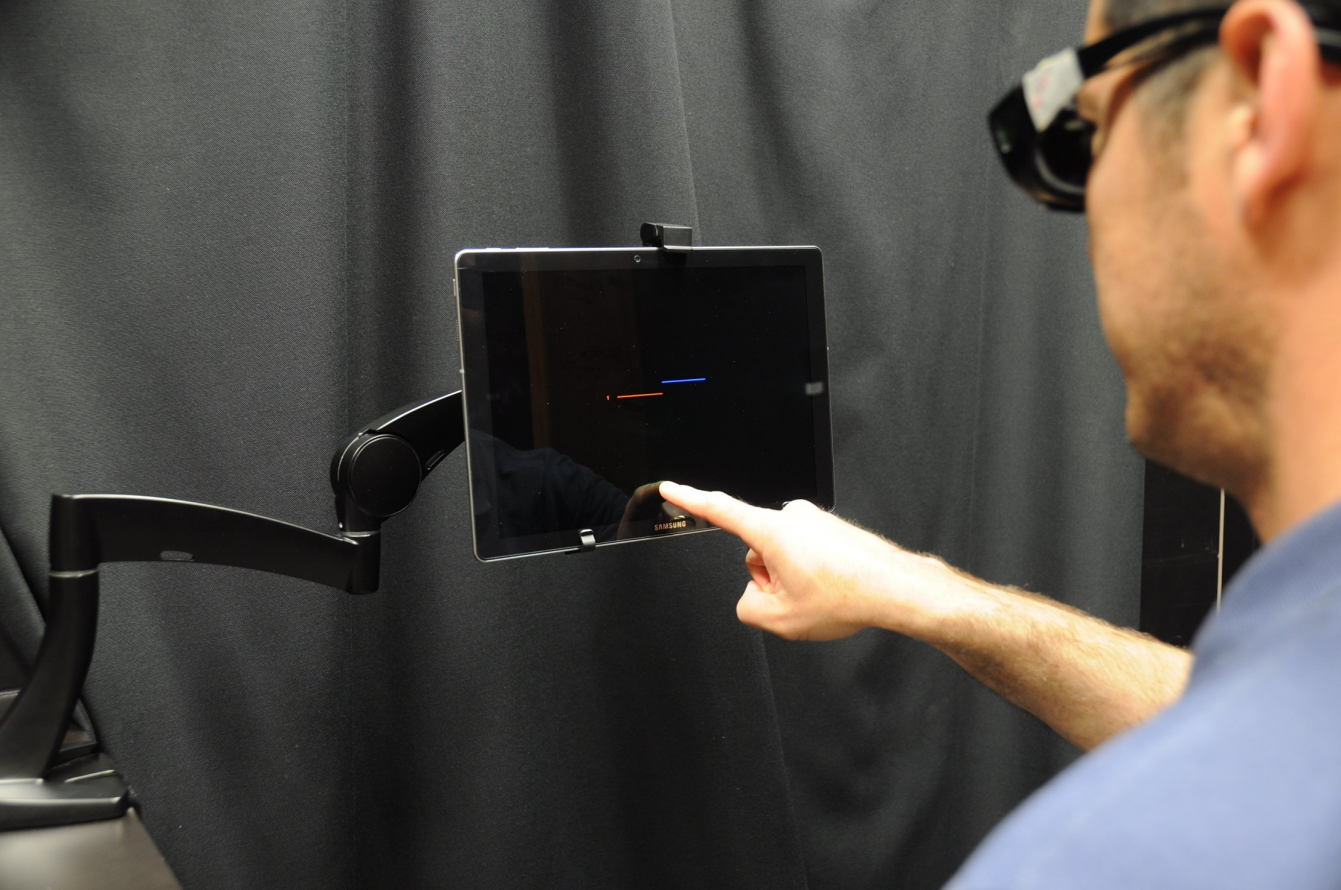
Vertical and torsional alignment nulling. The subject is seated 17.5” from a tablet positioned at eye level and mounted to an arm that is fixed to a table. At the start of each trial, the vertical position (VAN) or rotation angle (TAN) of the moving blue line is randomly positioned. Subjects then adjust the single blue line until it a continuous red-blue horizontal line is perceived. This is accomplished by dragging the finger along the tablet screen. The test should be done in complete dark. “This individual has given written informed consent (as outlined in PLOS consent form) to publish these case details”.

We have developed a behavioral measure of the two oculomotor signs reported as part of the OTR–skew deviation and roll tilt. Known as the Vertical and Torsional Alignment Nulling tests (VAN, TAN), subjects view a tablet computer that displays one red and one blue line while viewing through color-matched red and blue filters; this provides separate visual information from each eye to the brain and prevents binocular fusion One line, designated as the *stationary line*, remains fixed on the screen, while the other line, designated as the *moving line*, is repositioned by the subject vertically during VAN and rotationally during TAN (**Fig 1**). The subject’s task is to adjust the moving line until it appears perfectly in-line with the stationary line, in other words to null any apparent vertical or rotational offset between the two lines. The test has been validated using prism diopters and video-oculography as well as during the altered gravitational environment of parabolic flight [23, 24].The purpose of our study was to compare VAN and TAN between veterans and healthy controls in upright and supine test positions.

## Materials and methods

Veterans (n = 26) with reports of dizziness were recruited from the East Orange Veterans Affairs Hospital. Healthy controls (n = 26) were recruited from both Johns Hopkins University and the East Orange VA. All subjects signed consent forms approved by the local Institutional Review Board (East Orange VA and Johns Hopkins) as well as the Human Research Protection Office of the Department of Defense. The individual pictured in this manuscript has given written informed consent (as outlined in PLOS consent form) to publish these case details. Participants with hearing or visual impairment were excluded.

### Vestibular function testing

Each of the veterans underwent cervical and ocular vestibular evoked myogenic potential (VEMP) testing to measure otolith function, as well as video head impulse testing (vHIT) to measure the horizontal semicircular canal and afferent pathways. The cervical VEMP (cVEMP) test measures function from the inferior vestibular nerve and saccular otolith. The ocular VEMP (oVEMP) measures function from the superior vestibular nerve and utricular otolith. vHIT of the horizontal semicircular canals measures function from the superior vestibular nerve. VEMP testing was considered abnormal for reduced sound threshold (dB) and/or latency of the positive and negative response being greater than the mean and 2SD above age-matched controls [25].

### VAN and TAN

Every subject performed 20 trials each of VAN and TAN while seated in an upright position. The data was collected on an active-matrix organic LED (Samsung Galaxy TabPro S) tablet to ensure only the designated pixels on the tablet (i.e., the red and blue lines only) were illuminated. The tablet was fixed to an arm mounted to a table that was positioned 17.5” from lateral aspect of the subject’s eye. All subjects were trained on how to perform VAN and TAN prior to their data collection. Training included practicing the VAN and TAN tasks first in the light, in order to verify the test instructions were understood; training was repeated in complete darkness.

During testing, a trial counter is present for the subject and the software automatically switches to TAN testing after first completing 20 trials of VAN testing. If the subject enters an errant response, the trial can be deleted and then repeated. Measures collected are the amount in degrees by which the lines are separated from one another vertically (VAN) or rotated relative to one another (TAN), which provides a perceptual measure of vertical and torsional ocular misalignment, respectively. For example, if a subject orients the right line 5° counterclockwise relative to the left line during TAN, then we infer that this subject has a torsional misalignment such that the right eye is intorted 5° relative to the left eye. If a subject perfectly aligns the two lines during both VAN and TAN, then we infer that this individual has perfect vertical and torsional binocular alignment. For detail on the VAN and TAN method, please see Beaton et al. 2017 [24].

### Statistical analysis

The first ten trials each of VAN and TAN were processed for mean and SD values in upright and supine conditions (scores stabilize after ten trials) [26]. The data was not normally distributed hence we used the Mann-Whitney U non-parametric tests to evaluate for differences between the veterans and control groups. Additionally, we applied a log transformation to create a normalized distribution (i.e., SD of the 10 trials of the individuals VAN and TAN). vHIT data was analyzed using dependent T-test. All levels of statistical comparison were set at significance *p*≤0.05. SPSS (version 24, Chicago, Il, USA) was used to complete the statistical analysis.

### Demographic measures

The Posttraumatic Stress Disorder (PTSD) Checklist (PCL)-17 is a standardized self-report rating scale for Post-Traumatic Stress Disorder (PTSD) that consists of 17 items that correspond to the DSM III-R diagnostic criteria for PTSD. The usual cut point suggesting PTSD is 37 in veteran populations [27–29].

The Vestibular Disorders Activities of Daily Living Scale (VADL) assesses level of functional limitation or disability in people with vestibular disorders. Scores between 1 and 3 suggest greater independence [30].

The Vertigo Symptom Scale (VSS) is a 15 item self-report questionnaire assessing imbalance, somatic anxiety, and autonomic arousal. Severe dizziness is indicated for scores greater than 12 [31].

The Patient Health Questionnaire (PHQ) -8 is an eight-item self-report diagnostic and severity measure for depression. Major depressive disorders are considered for PHQ-8 scores ≥10 [32].

The Timed Up and Go test (TUG) quantifies the time taken by an individual to stand up from a standard armchair, walk a distance of 3 meters, turn 180 degrees, walk back to the chair, and sit down. Scores greater than 13.5 seconds indicate fall risk in older adults with vestibular disorders [33] scores greater than 11.1 seconds indicate increased fall risk in community dwelling elderly [34].

The DGI is an 8-item scale that determines fall risk in older adults. Scores <20 indicate increased risk of falling in older adults and those with vestibular disorders [35].

The Dizziness Handicap Inventory (DHI) measures a subject’s perception of how severe their dizziness is affecting their life. The index considers the emotional, physical, and functional aspects of their quality of life. For patients with peripheral or central pathological causes for dizziness, a total score greater than 16 indicates the responder believes the dizziness is of a significant handicap [36].

The Activities-specific Balance Confidence Scale (ABC) is a self-report measure that asks subjects to rate their confidence performing 16 activities of daily living [37]. ABC scores above 80 are indicative of high confidence in balance [38].

The 10-meter walk test measures gait speed. Healthy controls, matched in age (55–59 years) with our veteran population, walked a mean 1.4m/s [39].

The 2-minute walk test is a measure of endurance. Healthy controls, matched in age with our veteran population, walked a mean 191 meters [40].

## Results

Our healthy control subjects were of similar age (50.3 ± 17.3) with our veteran subjects (55.4 ± 11.5). Demographic descriptions categorizing the severity of injury, subjective scales rating their perception of disability and dizziness, and functional scales of balance were collected in the veteran subjects. Of the measures collected, PCL, DHI and the 2-minute walk test were all significantly abnormal (Table 1). Each veteran reported to have dizziness.

**Table 1:** Severity of injury, subjective ratings perception of disability and dizziness, and functional scales of balance in the Veterans.

**Physical Behavior Measures**					
**DGI**	**TUG (sec)**	***10M (m/s)***	***^2 min walk (m)***		
22.1 ± 2.2	9.7 ± 2.3	*1*.*5 ± 0*.*4*	*145*.*7 ± 21*		
**Categorization Measures**					
*PCL	**VADL**	**VSS**	**PHQ8**	**ABC**	^***a***^***DHI***
*40*.*4 ± 15*.*6*	2.1 ± 1.6	10.7 ± 10.4	7.6 ± 5.6	83.5 ± 13.9	*30*.*3 ± 28*.*1*

*Italics* denote abnormal score; PCL–measure of PTSD; VADL–Vestibular Disorders Activities of Daily Living; VSS–vertigo symptom scale; PHQ8 –measure of depression; ABC–activities-specific balance confidence scale; TUG–timed up and go; DGI–dynamic gait index; DHI–dizziness handicap inventory; M–meter

* scores > 37 are positive for suffering PTSD

^a^ scores > 16 significant for perceiving a handicap from dizziness

^ abnormal compared with age matched controls

### VEMP and vHIT testing

We collected cervical and ocular VEMP testing data on twenty of the 26 veterans (e.g., S1 Table). The other six veterans were unable to complete VEMP testing based on time constraints. Roughly one third of veterans had an absent cVEMP response (either right or left); another 1/3 had an inconclusive result. The final third of veterans subjects with a cVEMP response had mean (left and right) P1 latencies of 15.3 ± 1.72 ms and mean N1 latencies of 21.8 ± 1.6 ms. cVEMP responses were achieved at either the 93 or 97 dB threshold level. For oVEMP testing, more than half (~55%) had an absent response (either right or left) with the other 45% being either normal or inconclusive. The range of normal oVEMP scores were mean 15.3 ± 2.4 for P1 latency and 22.2 ± 2.ms for the N1 latency (left and right). Reasons for VEMP testing to be inconclusive include motion artifact or indeterminable waveforms.

We collected vHIT in 23 of the 26 veterans, as time availed. There was no difference in VOR gain between right (0.98 ± 0.12) and left (0.95 ± 0.1) passive yaw head rotations (p = 0.24).

### VAN and TAN

**Fig 2:**
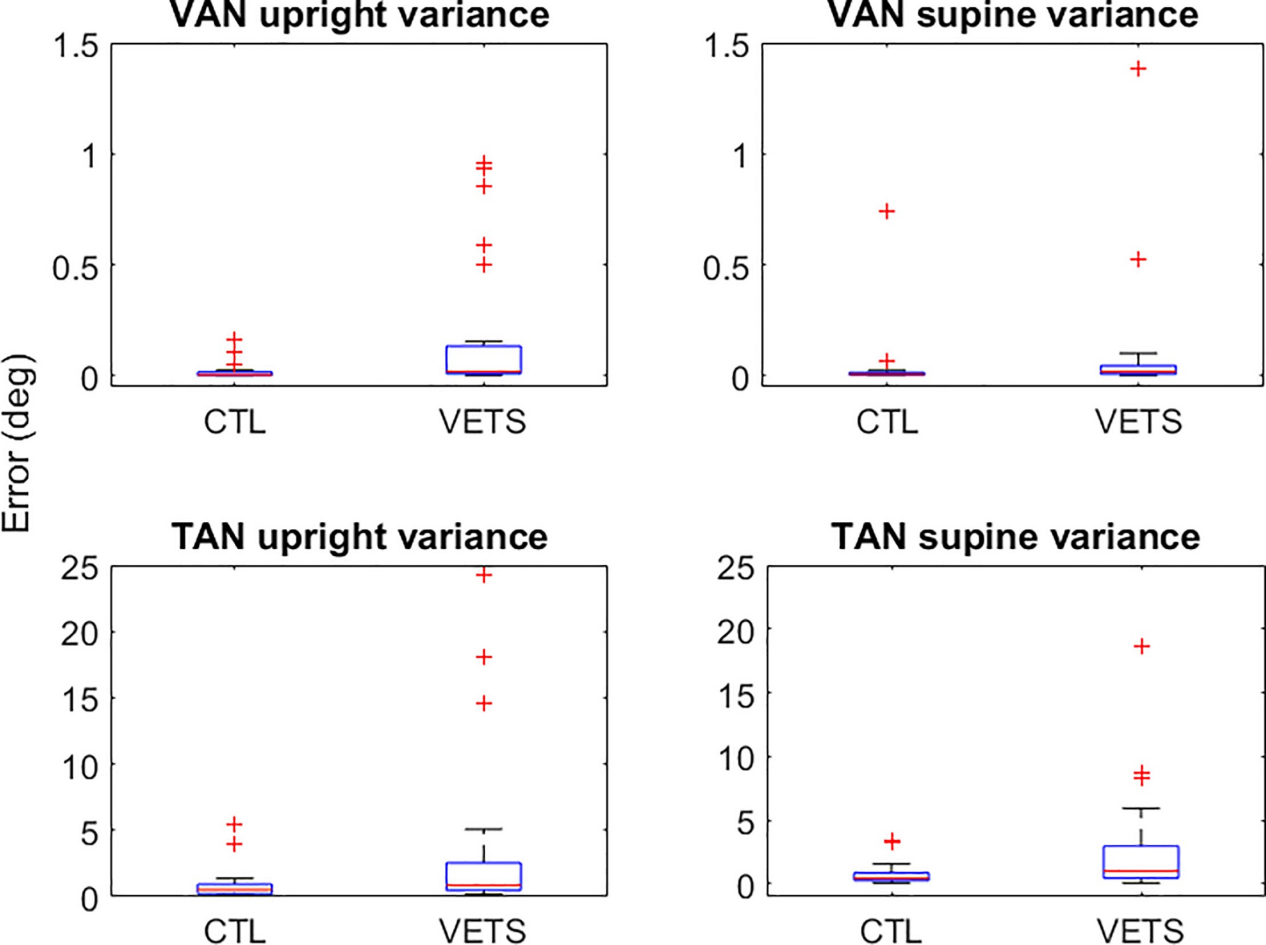
Box and whisker plot establishing greater within-subject variability of VAN and TAN scores during both upright sitting and supine test positions. CTL–Control; VETS–Veterans with dizziness.

Our data reveal that the Veterans have significantly greater ***within-subject variability***, in both their VAN and TAN scores for both upright and supine positions compared to the healthy controls. In the upright test position, the variability of VAN was 285% higher (p = 0.002) and the variability in TAN was 169% higher (p = 0.027) in the veterans compared with the controls. In supine, the variability was 201% higher for VAN (p = 0.009) and 170% greater (p = 0.022) for TAN compared with the controls (Fig 2).

We found no difference in the mean VAN or TAN scores between the veterans or controls in either upright or supine position (Table 2).

**Table 2:** Mean and one standard deviations (SD) of VAN and TAN for veterans and controls.

Group	VAN Up	VAN Supine	VAN Diff	TAN Up	TAN Supine	Tan Diff
**Veterans (n = 26)**	0.094 ± 0.23	0.277 ± 0.70	-0.175 ± 0.61	0.427 ± 0.92	0.241 ± 0.32	-0.047 ± 0.96
**Controls (n = 26)**	0.123 ± 0.37	0.157 ± 0.54	0.071 ± 0.20	0.275 ± 1.19	0.120 ± 0.15	0.777 ± 3.25

Diff–mean difference moving from Upright to Supine. Negative VAN values denote subjects adjusted the moving line higher than the stationary line. Negative TAN values denote subjects adjusted the moving line counter-clockwise to the stationary line. Veterans have significantly greater within-subject variability, in both their VAN and TAN scores for upright and supine position.

Sixteen of our 26 veteran subjects (62%) have MSI. However, we found no difference in VAN or TAN between those veterans with or without MSI (p ≥ 0.484). Fifteen (58%) of our veteran subjects have PTSD (PCL score 54.36 ± 10.72) while 11 (29.20 ± 7.60, p<0.001) did not, and there was no difference in VAN and TAN scores between the veterans with PTSD and those without (p ≥ 0.37).

## Discussion

Our data reveal that veterans with dizziness have a significantly increased variability for their perception of vertical and torsional ocular alignment (VAN and TAN). In contrast, the healthy control group showed a consistent performance in their measures of VAN and TAN. Furthermore, neither PTSD nor MSI were a contributor towards this increased variability in perception of oculomotor alignment. We are uncertain of the precise mechanism responsible for this increased variability of abnormal ocular misalignment although the added symptom of dizziness, common in each of the veteran subjects, entices us to posit an abnormal processing of ocular alignment via central vestibular or peripheral vestibular pathways given 88% of the veterans had an absent VEMP (cervical or ocular) response. Our data support healthy horizontal semicircular canal function [41], equating with the results from Akin et al [16] that showed a majority of injuries to service members affect the central or peripheral vestibular otolith end organ pathways. Another possible explanation for the increased variability of the veterans might be limited compensation reserves. Sadeghi et al. [42] showed that bilateral coordination during walking in healthy subjects is preserved even though asymmetries in muscle activity exist at the individual lower limb joints (knees and hips). This suggests that a neural mechanism compensates for the local asymmetries, thereby ensuring a global bilateral gait coordination. It is possible therefore, that by providing monocular visual cues (as we did by preventing fusion), we introduced a means that depleted their compensation reserves, impairing their perception of binocular alignment with a resultant increase in their variability.

To our knowledge, we are the first to report high variability in perception of binocular alignment for veterans with dizziness regardless of whether or not the veteran had a history of mTBI. This is a critical finding given symptoms of sensorimotor impairment may persist in the absence of subjective report [43]. Furthermore, we are not the first to show high variability in sensorimotor function in service members without TBI. Studying sub-concussive blast levels (blast waves below the known concussive thresholds) commonly experienced during heavy weapons training, Rhea et al [44] reported increased variability of stride time during “stepping-in-place” immediately after the training. The authors conclude that repeated exposure to low-level blast impairs neuromotor function. Others have shown patients with dizziness and TBI have increased variability in fall risk scores as measured by the dynamic gait index and Berg balance scale as well as worse static balance as measured from posturography [45]. Finally, those with a higher risk for falling (e.g. elderly) present with an increased variability of their gait pattern [46, 47]. Having sensitive tools onsite to accurately measure sensorimotor function in service members should be considered a high priority given that our data suggests veterans not only have impaired perception of binocular alignment regardless of having had a well-defined mTBI, but that this misalignment persists.

### Limitations

Our data should not extend to patient populations outside of veterans with dizziness. It remains possible that the veteran subjects may have some psychological confound (excluding PTSD) impacting their variability while performing the VAN and TAN tests–a confound that we did not measure. While PTSD was present in 58% of veterans, it had no effect on ocular misalignment. Depression scores in the veterans were not abnormal. Finally, it remains possible that our veteran subjects were exposed to multiple low-level blasts that impaired their perception of ocular alignment but did not otherwise impair their ability to perform their daily activities while on active duty.

## Conclusion

Our novel method of measuring vertical and torsional misalignment distinguishes veterans with dizziness from healthy controls. Further studies are needed to better understand the fundamental mechanism responsible for the increased variability of perception of binocular alignment, as well as to establish ranges of normal variability across unique patient populations.

## Supporting information

S1 Table Ocular and cervical vestibular evoked myogenic potential (Ovemp, Cvemp) data of n = 26 veteran subjects. (XLSX)

## References

[1] Cicerone KD, Kalmar K. Persistent postconcussion syndrome: The structure of subjective complaints after mild traumatic brain injury. J Head Trauma Rehabil. 1995;10(3):1–17.

[2] Roland PS, Haley RW, Yellin W, Owens K, Shoup AG. Vestibular dysfunction in gulf war syndrome. Otolaryngol Head Neck Surg. 2000;122:319–329. 10.1067/mhn.2000.105783 10699803

[3] Benge JF, Pastorek NJ, Thornton GM. Postconcussive symptoms in OEF-OIF veterans: factor structure and impact of posttraumatic stress. Rehabil Psychol. 2009;54(3):270–78. 10.1037/a0016736 19702425

[4] Zamyslowska-Szmytke E, Politanski P, Sliwinska-Kowalska M. Balance system assessment in workers exposed to organic solvent mixture. J Occup Environ Med. 2011 4;53(4):441–7. 10.1097/JOM.0b013e3182143f46 21407091

[5] Defense and Veterans Brain Injury Center. TBI facts Washington (DC): Department of Defense; 2009.

[6] Scott SG, Belanger HG, Vanderploeg RD, Massengale J, Scholten J. Mechanism-of-injury approach to evaluating patients with blast-related polytrauma. J Am Osteopath Assoc. 2006;106(5):265–70. 16717367

[7] Lew HL, Garvert DW, Pogoda TK, Hsu PT, Devine JM, White DK, Myers PJ, Goodrich GL. Auditory and visual impairments in patients with blast-related traumatic brain injury: Effect of dual sensory impairment on Functional Independence Measure. J Rehabil Res Dev. 2009;46(6):819–26. 20104405 10.1682/jrrd.2008.09.0129

[8] Lew HL, Pogoda TK, Baker E, Stolzmann KL, Meterko M, Cifu DX, Amara J, Hendricks AM. Prevalence of dual sensory impairment and its association with traumatic brain injury and blast exposure in OEF/OIF veterans. J Head Trauma Rehabil. 2011;26(6):489–96. 10.1097/HTR.0b013e318204e54b 21386715

[9] Rustemeyer J, Volker K, Bremerich A. Injuries in combat from 1982–2005 with particular reference to the head and neck: a review. Br J Oral Maxillofac Surg. 2007; 45:556–560. 10.1016/j.bjoms.2007.01.003 17316932

[10] Pogoda TK, Hendricks AM, Iverson KM, Stolzmann KL, Krengel MH, Baker E, Meterko M, Lew HL. Multisensory impairment reported by veterans with and without mild traumatic brain injury history. J Rehabil Res Dev. 2012;49(7):971–84. 23341273 10.1682/jrrd.2011.06.0099

[11] Warden D. Military TBI during the Iraq and Afghanistan wars. J Head Trauma Rehabil. 2006;21:398–402. 16983225 10.1097/00001199-200609000-00004

[12] Xydakis MS, Fravell MD, Nasser KE, Casler JD. Analysis of battlefield head and neck injuries in Iraq and Afghanistan. Otolaryngol Head Neck Surg. 2005;133:497–504. 10.1016/j.otohns.2005.07.003 16213918

[13] Defense Veterans Brain Injury Center. OIF/OEF Fact Sheet: June 2008 Washington, DC: Walter Reed Army Medical Center; 2008.

[14] Okie S. Traumatic brain injury in the war zone.N Engl J Med. 2005 5 19;352(20):2043–7. 10.1056/NEJMp058102 15901856

[15] http://www.va.gov/vetdata/docs/Quickfacts/Population_slideshow.pdf. Projected Veteran Population 2013 to 2043 Prepared by the National Center for Veterans Analysis and Statistics. October 13, 2014

[16] Akin FW, Murnane OD. Head injury and blast exposure: vestibular consequences. Otolaryngol Clin North Am. 2011 4;44(2):323–34. 10.1016/j.otc.2011.01.005 21474007

[17] Young ED, Fernandez C, Goldberg JM. Responses of squirrel monkey vestibular neurons to audio-frequency sound and head vibration. Acta Otolaryngol. 1977;84:352–360. 303426 10.3109/00016487709123977

[18] Halmagyi GM, Yavor RA, Colebatch JG. Tapping the head activates the vestibular system: a new use for the clinical reflex hammer. Neurology. 1995;45:1927–1929. 7477996 10.1212/wnl.45.10.1927

[19] Halmagyi GM, Gresty MA, Gibson WPR. Ocular tilt reaction with peripheral vestibular lesion. Ann Neurol 1979;6:80–83. 10.1002/ana.410060122 315752

[20] Brandt T, Dieterich M. Vestibular syndromes in the roll plane: Topographic diagnosis from brain stem to cortex. Ann Neurol 1994;36: 337–47. 10.1002/ana.410360304 8080241

[21] Brodsky MC, Donahue SP, Vaphiades M, Brandt T. Skew deviation revisited. Surv Ophthalmol 2006;51:105–28. 10.1016/j.survophthal.2005.12.008 16500212

[22] Wong AM, Colpa L, Chandrakumar M. Ability of an upright-supine test to differentiate skew deviation from other vertical strabismus causes. Arch Ophthalmol. 2011; 129(12):1570–5. 10.1001/archophthalmol.2011.335 22159676

[23] Beaton KH, Huffman WC, Schubert MC. Binocular misalignments elicited by altered gravity provide evidence for nonlinear central compensation. Front Syst Neurosci. 2015 6 2;9:81 10.3389/fnsys.2015.00081 26082691 PMC4451361

[24] Beaton KH, Shelhamer MJ, Roberts DC, Schubert MC. A rapid quantification of binocular misalignment without recording eye movements: Vertical and torsional alignment nulling. J Neurosci Methods. 2017 5 1;283:7–14. 10.1016/j.jneumeth.2017.03.009 28300605

[25] Nguyen KD, Welgampola MS, Carey JP. Test-retest reliability and age-related characteristics of the ocular and cervical vestibular evoked myogenic potential tests. Otol Neurotol. 2010 7;31(5):793–802. 10.1097/MAO.0b013e3181e3d60e 20517167 PMC2913294

[26] Schubert MC, Stitz J, Cohen HS, Sangi-Haghpeykar H, Mulavara AP, Peters BT, Bloomberg JJ. Prototype tests of vertical and torsional alignment nulling for screening vestibular function. J Vestib Res. 2017;27(2–3):173–176. 10.3233/VES-170618 29064832 PMC5659207

[27] Lang AJ, Laffaye C, Satz LE, Dresselhaus TR, Stein MB. Sensitivity and specificity of the PTSD checklist in detecting PTSD in female veterans in primary care. Journal of traumatic stress. 2003; 16(3):257–64. 10.1023/A:1023796007788 12816338

[28] Dobie DJ, Maynard C, Kivlahan DR, Johnson KM, Simpson T, David AC, et al Posttraumatic stress disorder screening status is associated with increased VA medical and surgical utilization in women. Journal of general internal medicine. 2006; 21 Suppl 3:S58±64. 10.1111/j.1525-1497.2006.00376.x . 16637948 PMC1513171

[29] Yeager DE, Magruder KM, Knapp RG, Nicholas JS, Frueh BC. Performance characteristics of the posttraumatic stress disorder checklist and SPAN in Veterans Affairs primary care settings. Gen Hosp Psychiatry. 2007; 29(4):294±301. 10.1016/j.genhosppsych.2007.03.004 17591505

[30] Cohen HS. Use of the Vestibular Disorders Activities of Daily Living Scale to describe functional limitations in patients with vestibular disorders. J Vestib Res. 2014;24(1):33–8. 10.3233/VES-130475 24594498

[31] Yardley L1, Masson E, Verschuur C, Haacke N, Luxon L. Symptoms, anxiety and handicap in dizzy patients: development of the vertigo symptom scale. J Psychosom Res. 1992 12;36(8):731–41. 1432863 10.1016/0022-3999(92)90131-k

[32] Kroenke K, Spitzer RL, Williams JB, Löwe B. The Patient Health Questionnaire Somatic, Anxiety, and Depressive Symptom Scales: a systematic review. Gen Hosp Psychiatry. 2010 Jul-Aug;32(4):345–59. 10.1016/j.genhosppsych.2010.03.006 20633738

[33] Whitney SL, Marchetti GF, Schade A, Wrisley DM. The sensitivity and specificity of the Timed "Up & Go" and the Dynamic Gait Index for self-reported falls in persons with vestibular disorders. J.Vestib.Res. 2004;14(5):397–409. 15598995

[34] Shumway-Cook A, Brauer S, Woollacott M. Predicting the probability for falls in community-dwelling older adults using the Timed Up & Go Test. Phys Ther. 2000;80(9):896–903. 10960937

[35] Shumway-Cook A, Baldwin M, Polissar NL, Gruber W. Predicting the probability for falls in community-dwelling older adults. Phys Ther. 1997;77(8):812–819. 9256869 10.1093/ptj/77.8.812

[36] Yorke A, Ward I, Vora S, Combs S, Keller-Johnson T. Measurement Characteristics and Clinical Utility of the Dizziness Handicap Inventory Among Individuals With Vestibular Disorders. Arch of Phys Med Rehab. Vol 94, Issue 11, 11 2013, Pages 2313–2314

[37] Powell LE, Myers AM. The Activities-specific Balance Confidence (ABC) Scale. J Gerontol A l Sci Med Sci. 1995;50A(1):28–34. 10.1093/gerona/50a.1.m28 7814786

[38] Myers AM, Fletcher PC. Discriminative and Evaluative Properties of the Activities-specific Balance Confidence (ABC) Scale. J. Gerontol Med Sci. 1998; 53A (4): M287–M294. 10.1093/gerona/53a.4.m287 18314568

[39] Bohannon RW and Williams AA. Normal walking speed: a descriptive meta-analysis. Physiotherapy. 2011 9;97(3):182–9. 10.1016/j.physio.2010.12.004 21820535

[40] Bohannon RW, Wang YC, Gershon RC. Two-minute walk test performance by adults 18 to 85 years: normative values, reliability, and responsiveness. Arch Phys Med Rehabil. 2015 3;96(3):472–7. 10.1016/j.apmr.2014.10.006 25450135

[41] Alshehri MM, Sparto PJ, Furman JM, Fedor S, Mucha A, Henry LC, et al The usefulness of the video head impulse test in children and adults post-concussion. J Vestib Res. 2016;26(5–6):439–446. 10.3233/VES-160598 28262647

[42] Sadeghi H. Local or global asymmetry in gait of people without impairments. Gait Posture. 2003 6;17(3):197–204. 12770633 10.1016/s0966-6362(02)00089-9

[43] Galea OA, Cottrell MA, Treleaven JM, O'Leary SP. Sensorimotor and Physiological Indicators of Impairment in Mild Traumatic Brain Injury: A Meta-Analysis. Neurorehabil Neural Repair. 2018;32(2):115–128. 10.1177/1545968318760728 29554850

[44] Rhea CK, Kuznetsov NA, Ross SE, Long B, Jakiela JT, Bailie JM, Yanagi MA, Haran FJ, Wright WG, Robins RK, Sargent PD, Duckworth JL. Development of a Por Tool for Screening Neuromotor Sequelae From Repetitive Low-Level Blast Exposure. Mil Med. 2017;182(S1):147–154. 10.7205/MILMED-D-16-00140 28291466

[45] Buster TW, Chernyavskiy P, Harms NR, Kaste EG, Burnfield JM. Computerized dynamic posturography detects balance deficits in individuals with a history of chronic severe traumatic brain injury. Brain Inj. 2016;30(10):1249–55. 10.1080/02699052.2016.1183822 27386896

[46] Guimaraes RM, Isaacs B. Characteristics of the gait in old people who fall. Int Rehabil Med. 1980;2(4):177–80. 7239777 10.3109/09638288009163984

[47] Hausdorff JM, Rios DA, Edelberg HK. Gait variability and fall risk in community-living older adults: a 1-year prospective study. Arch Phys Med Rehabil. 2001 8;82(8):1050–6. 10.1053/apmr.2001.24893 . 11494184

